# Network Pharmacology and Molecular Docking Analysis to Explore the Mechanism of Huaiqihuang-Mediated Alleviation of Henoch–Schönlein Purpura Nephritis

**DOI:** 10.1155/2022/2798217

**Published:** 2022-11-04

**Authors:** Qingqing Liu, Jiahua Liu, Yaya Du, Weiyan Guo, Jie Mi, Yanyan Guo

**Affiliations:** ^1^Precision Pharmacy & Drug Development Center, Department of Pharmacy, Tangdu Hospital, Air Force Medical University, Xi'an, Shaanxi 710038, China; ^2^Department of Clinical Laboratory, Xi'an Children's Hospital, Xi'an, Shaanxi 710003, China; ^3^Department of Pharmacy, The First Hospital of Xi'an, Xi'an, Shaanxi 710002, China; ^4^Department of Pharmacy, Xi'an Children's Hospital, Xi'an, Shaanxi 710003, China

## Abstract

**Objective:**

Henoch–Schönlein purpura nephritis (HSPN) is considered a major cause of chronic renal failure and is the most common secondary glomerular disease in children. Huaiqihuang (HQH), a traditional Chinese herbal formula, exhibits therapeutic effects against HSPN in clinical practice. However, the potential molecular targets and mechanisms underlying HSPN treatment remain unclear.

**Methods:**

By constructing a protein-protein interaction (PPI) network, core targets related to HQH and HSPN were identified. Gene Ontology enrichment and Kyoto Encyclopedia of Genes and Genomes pathways were analyzed to identify the main pathways related to HSPN based on the core targets. To screen the main active ingredients of HQH against HSPN, an ingredient-target-pathway network was constructed using the top 10 main pathways associated with HSPN. Then, molecular docking was performed to explore the interactions and binding patterns between molecules and proteins.

**Results:**

Clinical data showed that HQH combined with conventional medicine significantly reduced 24-hour urine protein excretion, urine microalbumin levels, and erythrocyte counts in the urine sediment of HSPN patients. By constructing PPI models, 15 potential core targets were identified. The top 10 main pathways showed higher enrichment ratios, including the cytokine–cytokine receptor interaction and signaling pathways related to NOD-like receptor, IL-17, etc. Through the ingredient-target-pathway network and molecular docking, we revealed that five active ingredients of HQH had good affinities with three core targets, AKT1, MMP9, and SERPINE1, which may be vital in treating HSPN.

**Conclusions:**

The study preliminarily explored the active ingredients, targets, and pathways involved in HQH therapy for HSPN. The mechanism of HQH therapy may be attributed to the modulation of inflammatory response, immune response, and oxidative stress. Combined with clinical data, our results indicate that HQH is highly effective in treating HSPN.

## 1. Introduction

In childhood, Henoch–Schönlein purpura (HSP) is a common vasculitis disease [1]. HSP can affect glomerular capillaries and interstitial blood vessels and cause kidney damage. Henoch–Schönlein purpura nephritis (HSPN) is the most severe complication of HSP. Approximately 30–50% children with HSP develop HSPN [2]. If HSPN is left untreated, it eventually leads to an unexpected chronic kidney disease in up to 20% of affected children [3], which can be further life-threatening.

Currently, glucocorticoid steroids combined with immunosuppressive therapy are recommended to treat HSPN [4]. However, clinical data suggest that this treatment has evident side effects such as gastrointestinal diseases, oncogenesis, and myelosuppression [5]. Therefore, it is necessary to identify new, effective, and safe strategies for the treatment of HSPN. In recent years, traditional Chinese medicine (TCM), including *Tripterygium* glycosides [6] and Huaiqihuang (HQH) [7], has shown prominent effects in preventing and treating HSPN.

HQH is a traditional Chinese herbal formula, which comprises *Trametes robiniophila* Murr (Huaier), *Polygonatum sibiricum* (HJ), and *Lycium barbarum* (GQZ). HQH has been used as an adjuvant for primary nephropathy, mycoplasma pneumonia, and bronchial asthma in children [5, 8]. HQH exhibits a significant therapeutic effect against kidney diseases by enhancing immune function and reducing proteinuria and hematuria [9, 10]. Moreover, HQH compensates for the defects of cyclophosphamide (CYP) by reducing nephrotoxicity [11]. However, our understanding of HQH in HSPN treatment remains unclear.

**Figure 1:**
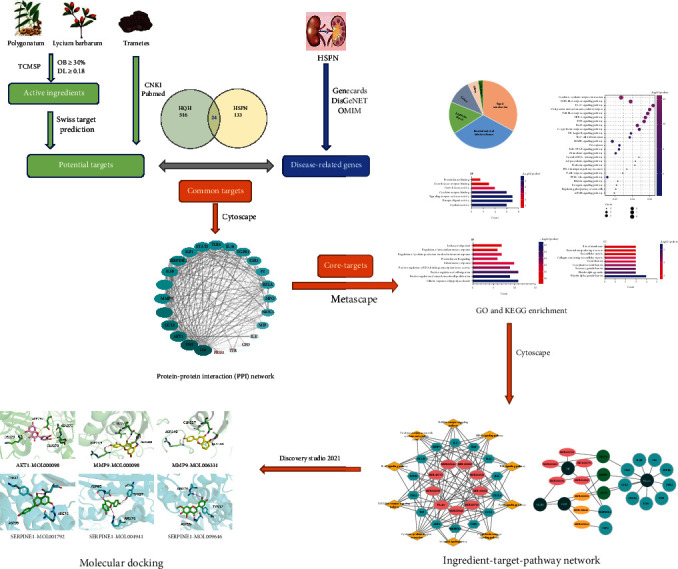
Flowchart of HQH in the treatment for HSPN.

It has been widely known that network pharmacology can explain the complex mechanisms and multiple effects of TCM [12]. Molecular docking is performed to predict the target proteins and active molecules. In the present study, we used network pharmacology and molecular docking to explore potential targets and molecules to understand the mechanism of action of HQH in HSPN treatment.  Figure 1 depicts the process flowchart in detail.

## 2. Methods

### 2.1. Analysis of HQH Clinical Efficacy on HSPN

This study included 30 children diagnosed with HSPN who underwent drug treatment in the Xi'an Children's Hospital from June 2021 to December 2021. All data were sourced retrospectively from the hospital information system. The control group consisted of 15 children who received conventional treatment with an oral prednisone tablet and an intravenous infusion of CYP. The remaining 15 children in the experimental group were administered HQH granules combined with the conventional treatment. Renal function indicators, including 24-hour urine protein excretion, urinary microalbumin, and erythrocyte count in urine sediment, were assessed before and after 3-month treatment. SPSS 20.0 software was used for data processing and analysis.

The inclusion criteria were as follows: (1) Clinical Guidelines for Nephrology [13] was used for diagnosis of HSPN; (2) all cases and laboratory data were available; and (3) the patients showed good compliance.

The exclusion criteria were as follows: (1) glomerulonephritis, IgA nephropathy, and other kidney diseases; (2) allergic to the medicine during treatment; and (3) patients with other autoimmune diseases.

The outcome was measured based on (1) 24-hour urine protein excretion, (2) urinary microalbumin content, and (3) erythrocyte count in urine sediment.

### 2.2. Search and Collection of Active Ingredients of HJ and GQZ

The Chinese State Food and Drug Administration has authorized the application of Huaier granules to treat various cancers. Evidence showed that proteoglycans were active ingredients of Huaier [14]. The active ingredients of HJ and GQZ were searched in the Traditional Chinese Medicine Systems Pharmacology (TCMSP) database (http://tcmspw.com/tcmsp.php) conforming to oral bioavailability ≥ 30% and drug‐likeness ≥ 0.18 [15]. The structure-data file (SDF) format of all active ingredients was downloaded from the PubChem database (https://pubchem.ncbi.nlm.nih.gov/) for subsequent prediction of potential targets and molecular docking.

### 2.3. Identification of the Active Ingredient Targets and Disease-Related Genes

The SDF files of active ingredients were imported into the Swiss Target Prediction database (http://www.swisstargetprediction.ch) to obtain active ingredient targets. Since the targets for Huaier could not be obtained here, we searched and collected them from PubMed and China National Knowledge Infrastructure (CNKI) databases.

Disease-related genes were obtained through GeneCards (https://www.genecards.org/), OMIM (https://www.omim.org/), and DisGeNET (http://www.disgenet.org) with the keywords “Henoch–Schönlein purpura nephritis.” The genes were limited to “*Homo sapiens*.” All genes were downloaded and integrated using Microsoft Excel.

### 2.4. Protein-Protein Interaction (PPI) Network

Venn diagrams (https://bioinfogp.cnb.csic.es/tools/venny_old/) were generated to determine the common targets between HQH and disease. Then, the PPI network of common targets was constructed by the STRING database (https://string-db.org/). “*Homo sapiens*” was chosen, and a medium confidence score > 0.4 was selected. The above results were imported into Cytoscape (v3.7.2) (https://cytoscape.org/) for visualization. These targets were defined as core targets if their degree value exceeded the average.

### 2.5. Gene Ontology (GO) and Kyoto Encyclopedia of Genes and Genomes (KEGG) Pathway Enrichment Analysis

The Metascape database (http://metascape.org/) was used to perform GO and KEGG analysis. The core targets were imported, and “*Homo sapiens*” was selected. An online bioinformatics web (http://www.bioinformatics.com.cn) was used to visualize the results. The cutoff chosen for the *p* value was <0.01.

### 2.6. Molecular Docking

Molecular docking was carried out using Discovery Studio 2021 to explore the interactions and binding patterns between molecules and proteins. The active ingredients (ligands) of HQH were stored as SDF files. The main protein targets were obtained from the Protein Data Bank (http://rcsb.org/). Additionally, the best protein crystal structure was characterized based on the images with a low resolution with observable ligands and a relatively intact structure. The ligands were prepared using the Ligand Preparation module and minimized using the CHARM force field to generate 10 conformers. Using the Receptor-Ligand Pharmacophore Generation module to define the ligand-binding site, original ligands were extracted from the target proteins. The docking results were evaluated based on -CDOCKER energy, hydrogen bond interaction, and the pattern of binding mode.

## 3. Results

### 3.1. HQH Enhanced Conventional Medicine Efficacy for Treating HSPN Children

In our study, the experimental group consisted of eight boys and seven girls aged between 3.75 and 13.33 (8.15 ± 2.7) years. The control group consisted of ten boys and five girls, and their ages ranged from 4.5 to 12.25 (9.03 ± 2.28) years. The degree of renal function damage is a prognostic factor for HSPN, as HSPN manifestation is primarily marked by abnormal urine, including a decrease in proteinuria and hematuria. In clinics, 24-hour urine protein excretion, urine microalbumin, and erythrocyte counts in urine sediment are used as renal function indices. Therefore, we used these three indices to evaluate the therapeutic effect of HQH in this study. Before treatment, the groups showed no significant differences in 24-hour urine protein excretion, urine microalbumin levels, and erythrocyte counts in urine sediment (*p* > 0.05). However, all indices significantly decreased after 3-month treatment in both groups (*p* < 0.05). Moreover, the experimental group showed indices lower than the control group (*p* < 0.05) (Table 1). Therefore, HQH was effective as an adjuvant for treating HSPN.

### 3.2. Identification of Active Ingredients of HQH and the Corresponding Targets

The total number of active ingredients selected from GQZ and HJ was 45 and 12, respectively (Table 2). The Swiss Target Prediction database suggested more than 524 targets of GQZ and HJ, and 482 targets related to GQZ-HJ were selected after removing duplicates for further studies. Another component of HQH is Huaier, which is a fungus. The PubMed and CNKI databases suggested 73 valid targets of Huaier. Taken together, we identified 540 HQH targets for further study.

### 3.3. Identification of Target Genes of the Diseases

We searched for HSPN-related genes using GeneCards, DisGeNET, and OMIM and identified 84, 29, and 63 HSPN-related genes, respectively. After removing the duplicate genes and integrating the results, 157 candidate genes were identified.

### 3.4. Acquiring the Core Targets by PPI Network

**Figure 2:**
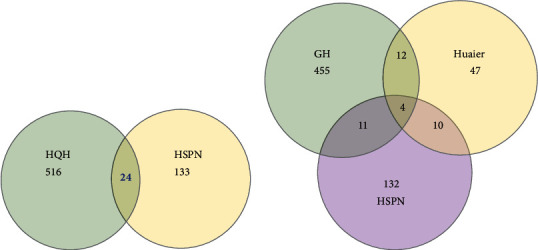
Common targets. (a) Identification of common targets between HQH and HSPN; (b) identification of common targets between HSPN, Huaier, and GQZ-HJ (GH).

To obtain the potential key genes targeted by HQH during HSPN treatment, we considered the overlap of HQH targets and disease-related genes and identified 24 common targets correlated with HSPN (Figure 2), including AKT1, CCL2, CXCL8, ESR1, IL10, IL18, IL6, STAT3, RELA, IL1*β*, TLR4, TNF, TGFB1, CFD, F2, IGF1, ILK, MIF, MMP9, MPO, NR3C1, PRSS1, SERPINE1, and TTR. Importantly, AKT1, MMP9, ESR1, and STAT3 were correlated with Huaier, GQZ-HJ, and HSPN.

**Figure 3:**
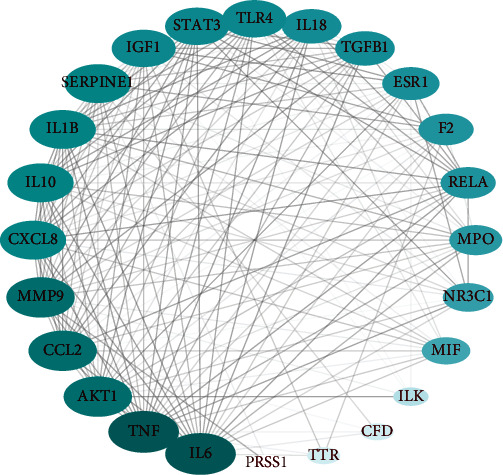
Protein-protein interaction (PPI) analysis of common targets.

The degree parameter was calculated to analyze the importance of common targets in the PPI network using Cytoscape software. A total of 24 nodes and 179 edges were visualized (Figure 3). Larger size and darker color indicate a higher degree of nodes, indicating a stronger interaction with other targets. All targets with degree values greater than the average were considered core targets (average degree = 14.90). The core targets related to HSPN are shown in Table 3, which include IL6, TNF, AKT1, CCL2, MMP9, SERPINE1, IL1B, CXCL8, IL10, STAT3, IGF1, TLR4, TGFB1, IL18, and ESR1.

### 3.5. KEGG Pathway and GO Analysis

**Figure 4:**
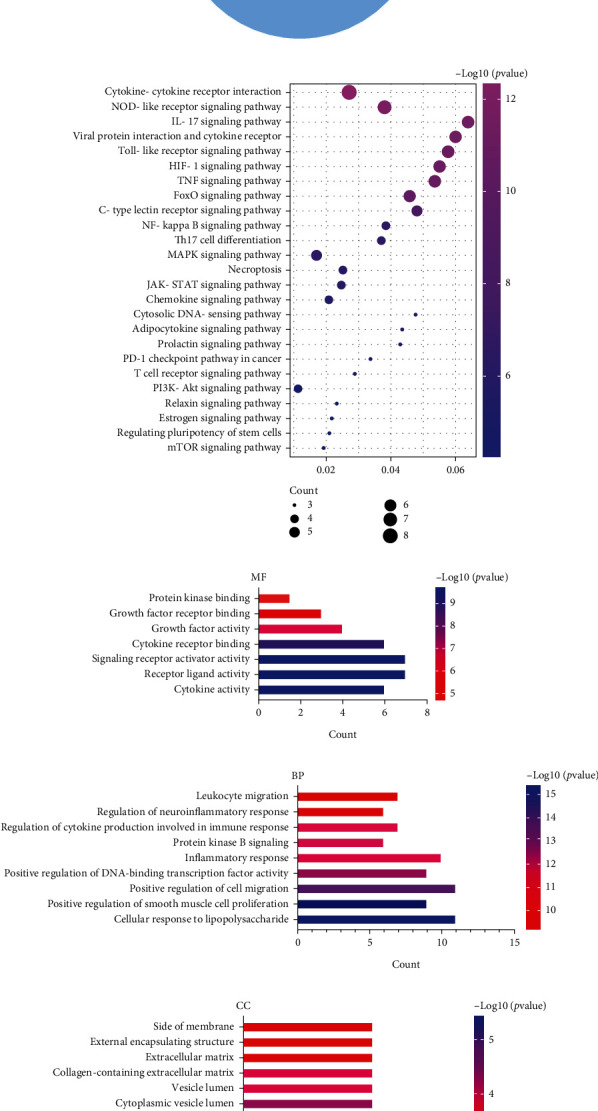
KEGG pathway and GO enrichment analyses.

In total, 79 KEGG pathways were significantly enriched. These pathways were mainly related to bacterial and viral diseases and signal transduction pathways (Figure 4(a)). Because we aimed to explore the potential biological mechanism of HSPN, pathway terms directly related to other diseases and different functional categories were removed. The HSPN-related signal transduction pathways were further analyzed and are illustrated in Figure 4(b). Compared with other pathways, the top 10 main pathways are presented in Table 4.

GO analysis was performed to explore the molecular functions, biological processes, and cellular components of the core targets (Figures 4(c)–4(e)). By setting the filter as a *p* value cutoff < 0.01, the molecular function of 15 core targets primarily focused on cytokine activity, receptor-ligand interaction, activity of growth factors, and growth factor-receptor binding. The biological processes of HSPN mainly involve the inflammatory response, regulation of cell migration, regulation of cell proliferation and apoptosis by modulating the cellular response to lipopolysaccharide, leukocyte migration, and regulation of interleukin-6 production. Cellular components were related to the side of the membrane, extracellular matrix, vesicle lumen, and platelet alpha granules. In summary, we found that the 15 core targets were enriched in signaling pathways and biological mechanisms associated with inflammation and immune response.

### 3.6. Construction of the Ingredient-Target-Pathway Network

**Figure 5:**
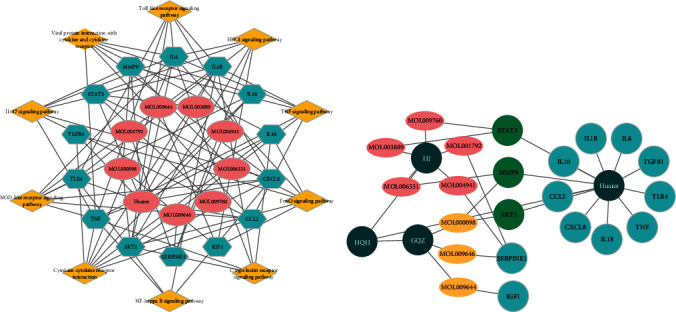
(a) Ingredient-target-pathway network; (b) ingredient-target network.

To understand which ingredient regulates these core targets for treating HSPN through the top 10 main pathways, we constructed the ingredient-target-pathway interaction network using Cytoscape. ESR1 was excluded from the network because it was not enriched in these pathways. The remaining 14 core targets were identified by Huaier and different active ingredients from HJ and GQZ, including MOL003889, MOL009760, MOL000098, MOL001792, MOL004941, MOL009646, MOL009644, and MOL006331 (Figure 5(a)). Finally, we screened eight active ingredients from this network for further analysis.

Furthermore, we found that MOL003889, MOL009760, MOL006331, MOL001792, and MOL004941 were correlated to HJ, and MOL009646, MOL009644, and MOL000098 were associated with GQZ (Figure 5(b)). Our results showed that Huaier mainly acted on targets associated with immunity and inflammation, such as IL6, IL10, IL1*β*, TLR4, and CCL2. The three common targets, AKT1, MMP9, and STAT3, were modulated by Huaier, MOL000098, MOL006331, MOL003889, and MOL009760. In addition, MOL001792, MOL004941, MOL009646, and MOL009644 interacted with SERPINE1 and IGF1. Therefore, Huaier and active ingredients from HJ and GQZ may be utilized to treat HSPN by regulating their respective targets.

### 3.7. Molecular Docking

To verify whether the eight active ingredients could interact with targets, we used molecular docking to analyze their binding mode and affinity. We set the top hit to 10 and pose cluster radius to 0.5 to ensure as diverse docking conformations as possible. A high -CDOCKER energy indicates a high affinity between the molecules and protein receptors. We selected ingredients with lower -CDOCKER energy than that of the original ligand and the target protein as potentially active molecules. Except for STAT31 and IGF1, other active ingredients showed good affinity to the targets. Five bioactive molecules were screened based on their energy, as shown in Table 5.

**Figure 6:**
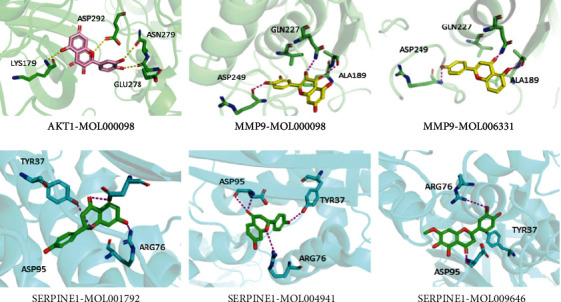
Docking results of active ingredients with target proteins.

Among these, MOL000098 had the highest affinity for AKT1. MOL000098 interacted with Lys179, Asp292, Asn279, and Glu278 via hydrogen bonding and entered the active pocket of AKT1 (Figure 6). MOL000098 and MOL006331 fit well within the active pocket of MMP9 and interacted with Ala189, Gln227, and Asp249 to form hydrogen bonds. MOL001792, MOL004941, and MOL009646 occupied the active pocket of SERPINE1 and formed stable hydrogen bonds with Asp95, Arg76, and Tyr37. The names and structures of the potentially active ingredients are listed in Table 6.

## 4. Discussion

HQH is effective in treating HSPN. However, its active ingredients and underlying biological mechanisms are unclear. Our study prospectively predicts the bioactive ingredients and potential targets of HQH for HSPN treatment from the perspective of network pharmacology. We found that HQH improved kidney function indices to enhance the efficacy of conventional medicine in treating children with HSPN. Second, network pharmacology demonstrated that HQH affected 15 core targets related to HSPN. Subsequently, GO and KEGG enrichment analyses showed that these core targets may participate in the immune and inflammatory responses and oxidative stress. Finally, molecular docking defined suitable active ingredients and potential target proteins. Together, our results suggest that the five ingredients from HJ and GQZ could treat HSPN by regulating AKT1, MMP9, and SERPINE1 with the exception of Huaier.

HSPN is a self-limiting immune disease, which can cure spontaneously or lead to chronic kidney disease. Our results showed that HQH combined with conventional medicine significantly reduced 24-hour urine protein excretion, urine microalbumin levels, and erythrocyte counts in urine sediment. Therefore, HQH can alleviate kidney damage and enhance the effect of conventional medicines on HSPN. In addition, conventional medicines, such as CYP, increase nephrotoxicity while treating HSPN [16]. Importantly, HQH has a protective effect against CYP-induced nephrotoxicity [11]. Therefore, HQH may be a special adjunctive drug in treating HSPN.

HQH is a Chinese herbal compound comprising Huaier, GQZ, and HJ. Huaier has demonstrated promising curative effects in clinical treatment for various cancers [17]. The anticancer activity of Huaier results from the action of its polysaccharides [18] that show antitumor and immunomodulatory effects [14]. Huaier protects the kidney and relieves nephrotoxicity by reducing oxidative stress and inflammation, promoting the recovery of mitochondrial function and inhibiting the NF-*κ*B signaling pathway [19]. GQZ and HJ, the main ingredients of HQH, have been extensively used to treat various chronic kidney diseases. However, the specific roles of GQZ and HJ in treating HSPN are yet unknown.

Consistent with previous results, our study showed that Huaier mainly regulated immune-related targets (IL6, TNF, CCL2, IL1B, CXCL8, IL10, TLR4, TGFB1, and IL18). MOL000098 and MOL006331 could interact with AKT1 and MMP9. MOL009646 from GQZ and MOL001792 and MOL009641 from HJ could bind to SERPINE1. These components may play vital roles in HSPN treatment.

Immune abnormalities and inflammatory injuries are involved in the pathogenesis of HSPN [20]. Children with HSPN show an imbalance in Th17/Treg cells [21, 22]. IL-6 is an important cytokine in Th17 cells. Treg cells exert their effects via TGF-*β* and IL-10. Patients with HSPN present high levels of IL-8, CCL2, TNF-*α*, IL-10, and IL-6 [23, 24]. HQH administration elevates Treg and lowers Th17 levels [5, 25]. Consistent with previous results, our study showed that Huaier acts on IL6, CCL2, IL-10, and TNF. These targets are covered by signaling pathway related to NOD-like receptor and IL-17, viral protein interaction with the cytokine and cytokine receptor, and cytokine-cytokine receptor interaction. An important role for STAT3 is to regulate antitumor immunity response. STAT3 activation can produce various immunosuppressive factors such as IL-6, IL-10, TGF*β*, and CCL2 [26]. Previous studies have shown that Huaier can interact with STAT3 [27]. Unexpectedly, we observed a poor binding between HJ (MOL003889 and MOL009760) and STAT3.

AKT1 (also known as mitochondrial protein kinase B) indirectly prevents the development of glomerulosclerosis and subsequent chronic kidney disease [28]. We observed that MOL000098 and Huaier interacted to stabilize AKT1. MMP9 controls developmental processes, tissue remodeling, and inflammatory responses [29]. MMP9 has been recognized in chronic kidney diseases and is an important indicator for the early diagnosis of HSPN [30]. In our study, Huaier, MOL000098 (GQZ), and MOL006331 (HJ) regulated MMP9 expression. In comparison with the control groups, the level of IGF-1 in HSPN children was significantly increased [31]. Unfortunately, MOL009644 had poor binding energy with IGF-1. SERPINE1, also known as plasminogen activator inhibitor type 1 (PAI-1), is produced in small amounts in healthy kidneys but is highly expressed in chronic kidney diseases [32]. SERPINE1 might be used as a potential biomarker for the pathology and progression of kidney diseases [33]. We found that SERPINE1 expression was regulated by HJ and GQZ. Altogether, our study indicates that HQH can be effective in treating HSPN through multiple important targets.

Oxidative stress pathologically aggravates kidney diseases. HQH can alleviate oxidative damage [11]. Moreover, HQH relieves CYP-induced kidney damage by suppressing the MAPK/NF-*κ*B pathway, which is associated with oxidative stress regulation. In the present study, the NF-*κ*B pathway was regulated by IL1B, CXCL8, TLR4, and TNF. In addition, HIF-1 activation is closely related to inflammatory responses, while FOXO can regulate oxidative stress, autophagy, and apoptosis. Both HIF-1 and FOXO were covered by core genes from HQH. Taken together, HQH exhibits significant antioxidant effects, reduces inflammatory damage, and improves humoral immunity.

## 5. Conclusions

HQH plays an important role in improving HSPN and shows promising therapeutic efficacy by modulating inflammatory response, immune response, and oxidative stress. In this study, the clinical application evidence of HQH in treating HSPN is provided. Furthermore, some shortcomings remain in the study. The prediction of the ingredients, targets, and pathways still needs further pharmacological experimental verification.

## Figures and Tables

**Table 1:** HQH enhanced efficacy of conventional drugs by improving renal function indices.

Group	Case number	Time	24-hour urine protein excretion (mg/24 h)	Urinary microalbumin (mg/L)	Erythrocyte count in urine sediment (Pcs/*μ*L)
Experimental group	15	Before	1580.21 ± 670	62.81 ± 24.45	99.34 ± 40.11
		After	291.40 ± 194.41^∗^^#^	11.64 ± 5.6^∗^^#^	5.24 ± 2.71^∗^^#^
Control group	15	Before	1593.96 ± 682.22	62.2 ± 21.32	120.05 ± 38.52
		After	472.76 ± 191.28^∗^	18.35 ± 9.12^∗^	10.55 ± 5.74^∗^

^∗^After 3-month treatment compared with before treatment in both the experimental and control groups, *p* < 0.05. ^#^The experimental group compared with the control group after 3-month treatment, *p* < 0.05.

**Table 2:** Active ingredients in GQZ and HJ.

Drug source	Molecule ID	Active ingredients	OB (%)	DL
GQZ	MOL001323	Sitosterol alpha 1	43.28	0.78
	MOL003578	Cycloartenol	38.69	0.78
	MOL001494	Mandenol	42	0.19
	MOL001495	Ethyl linolenate	46.1	0.2
	MOL001979	LAN	42.12	0.75
	MOL000449	Stigmasterol	43.83	0.76
	MOL000358	Beta-sitosterol	36.91	0.75
	MOL005406	Atropine	45.97	0.19
	MOL005438	Campesterol	37.58	0.71
	MOL006209	Cyanin	47.42	0.76
	MOL007449	24-Methylidenelophenol	44.19	0.75
	MOL008173	Daucosterol_qt	36.91	0.75
	MOL008400	Glycitein	50.48	0.24
	MOL010234	Delta-carotene	31.8	0.55
	MOL000953	CLR	37.87	0.68
	MOL009604	14b-Pregnane	34.78	0.34
	MOL009612	(24R)-4Alpha-methyl-24-ethylcholesta-7,25-dien-3beta-ylacetate	46.36	0.84
	MOL009615	24-Methylenecycloartan-3beta,21-diol	37.32	0.8
	MOL009617	24-Ethylcholest-22-enol	37.09	0.75
	MOL009618	24-Ethylcholesta-5,22-dienol	43.83	0.76
	MOL009620	24-Methyl-31-norlanost-9(11)-enol	38	0.75
	MOL009621	24-Methylenelanost-8-enol	42.37	0.77
	MOL009622	Fucosterol	43.78	0.76
	MOL009631	31-Norcyclolaudenol	38.68	0.81
	MOL009633	31-Norlanost-9(11)-enol	38.35	0.72
	MOL009634	31-Norlanosterol	42.2	0.73
	MOL009635	4,24-Methyllophenol	37.83	0.75
	MOL009639	Lophenol	38.13	0.71
	MOL009640	4Alpha,14alpha,24-trimethylcholesta-8,24-dienol	38.91	0.76
	MOL009641	4Alpha,24-dimethylcholesta-7,24-dienol	42.65	0.75
	MOL009642	4Alpha-methyl-24-ethylcholesta-7,24-dienol	42.3	0.78
	MOL009644	6-Fluoroindole-7-dehydrocholesterol	43.73	0.72
	MOL009646	7-O-Methylluteolin-6-C-beta-glucoside_qt	40.77	0.3
	MOL009650	Atropine	42.16	0.19
	MOL009651	Cryptoxanthin monoepoxide	46.95	0.56
	MOL009653	Cycloeucalenol	39.73	0.79
	MOL009656	(E,E)-1-Ethyl octadeca-3,13-dienoate	42	0.19
	MOL009660	Methyl (1R,4aS,7R,7aS)-4a,7-dihydroxy-7-methyl-1-[(2S,3R,4S,5S,6R)-3,4,5-trihydroxy-6-(hydroxymethyl)oxan-2-yl]oxy-1,5,6,7a-tetrahydrocyclopenta[d]pyran-4-carboxylate	39.43	0.47
	MOL009662	Lantadene A	38.68	0.57
	MOL009664	Physalin A	91.71	0.27
	MOL009665	Physcion-8-O-beta-D-gentiobioside	43.9	0.62
	MOL009677	Lanost-8-en-3beta-ol	34.23	0.74
	MOL009678	Lanost-8-enol	34.23	0.74
	MOL009681	Obtusifoliol	42.55	0.76
	MOL000098	Quercetin	46.43	0.28

HJ	MOL001792	DFV	32.76	0.18
	MOL002714	Baicalein	33.52	0.21
	MOL002959	3′-Methoxydaidzein	48.57	0.24
	MOL000358	Beta-sitosterol	36.91	0.75
	MOL000359	Sitosterol	36.91	0.75
	MOL003889	Methylprotodioscin_qt	35.12	0.86
	MOL004941	(2R)-7-Hydroxy-2-(4-hydroxyphenyl)chroman-4-one	71.12	0.18
	MOL000546	Diosgenin	80.88	0.81
	MOL006331	4′,5-Dihydroxyflavone	48.55	0.19
	MOL009760	Sibiricoside A_qt	35.26	0.86
	MOL009763	(+)-Syringaresinol-O-beta-D-glucoside	43.35	0.77
	MOL009766	Zhonghualiaoine 1	34.72	0.78

**Table 3:** Information of core targets from HQH for HSPN treatment.

No.	Name	Degree	No.	Name	Degree
1	IL6	21	13	TGFB1	16
2	TNF	21	14	IL18	16
3	AKT1	20	15	ESR1	15
4	CCL2	20	16	F2	14
5	MMP9	20	17	RELA	14
6	SERPINE1	19	18	MPO	13
7	IL1B	19	19	NR3C1	12
8	CXCL8	19	20	MIF	11
9	IL10	19	21	ILK	5
10	STAT3	18	22	CFD	4
11	IGF1	18	23	TTR	4
12	TLR4	18	24	PRSS1	2

**Table 4:** Top 10 KEGG pathway analyses for the treatment of HSPN.

Rank	Pathways	*p* value	Enrichment	Targets
1	Cytokine-cytokine receptor interaction	4.57 × 10^−13^	0.027119	IL1B, IL6, CXCL8, IL10, IL18, CCL2, TGFB1, TNF
2	NOD-like receptor signaling pathway	1.72 × 10^−12^	0.038043	IL1B, IL6, CXCL8, IL18, CCL2, TLR4, TNF
3	IL-17 signaling pathway	3.78 × 10^−12^	0.06383	IL1B, IL6, CXCL8, MMP9, CCL2, TNF
4	Viral protein interaction with cytokine and cytokine receptor	5.53 × 10^−12^	0.06	IL6, CXCL8, IL10, IL18, CCL2, TNF
5	Toll-like receptor signaling pathway	7.04 × 10^−12^	0.057692	AKT1, IL1B, IL6, CXCL8, TLR4, TNF
6	HIF-1 signaling pathway	9.38 × 10^−12^	0.055046	AKT1, IGF1, IL6, SERPINE1, STAT3, TLR4
7	TNF signaling pathway	1.11 × 10^−11^	0.053571	AKT1, IL1B, IL6, MMP9, CCL2, TNF
8	FoxO signaling pathway	2.88 × 10^−11^	0.045802	AKT1, IGF1, IL6, IL10, STAT3, TGFB1
9	C-type lectin receptor signaling pathway	1.28 × 10^−9^	0.048077	AKT1, IL1B, IL6, IL10, TNF
10	NF-*κ*B signaling pathway	1.76 × 10^−7^	0.038462	IL1B, CXCL8, TLR4, TNF

**Table 5:** Molecular docking score.

Gene	PDB	Molecular	-CDOCKER energy
STAT3	6NJS	MOL003889	−66.47
		MOL009760	−64.40

AKT1	3OCB	MOL000098	49.06

MMP9	6ESM	MOL006331	34.22
		MOL000098	39.64

SERPINE1	4AQH	MOL001792	30.74
		MOL004941	30.14
		MOL009646	41.10

IGF1	5HZN	MOL009644	−40.60

**Table 6:** Potential active ingredients of HQH.

Sources	Mol ID	Molecule name	Structure
HJ	MOL001792	DFV	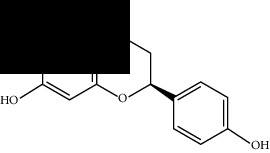
	MOL006331	4′,5-Dihydroxyflavone	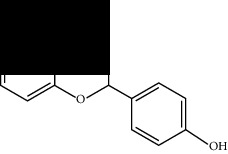
	MOL004941	(2R)-7-Hydroxy-2-(4-hydroxyphenyl)chroman-4-one	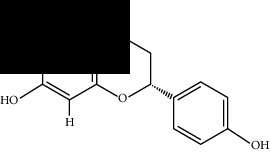

GQZ	MOL000098	Quercetin	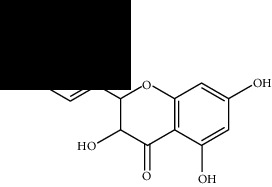
	MOL009646	7-O-Methylluteolin-6-C-beta-glucoside_qt	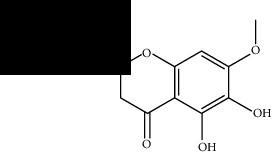

## Data Availability

The data of this research is obtained through authoritative online databases and software analysis and can be acquired from this study and supplementary material (available here).

## Supplementary Materials

Supplementary Materials Table 1: references of Huaier's targets. Table 2: targets of Huaier, GQZ-HJ, HQH, and HSPN.
