# Supplementary material for: Network Pharmacology and Molecular Docking Analysis to Explore the Mechanism of Huaiqihuang-Mediated Alleviation of Henoch–Schönlein Purpura Nephritis
Source: Biomed Res Int. 2022 Nov 4;2022:2798217. doi: 10.1155/2022/2798217 (PMC9652090; doi:10.1155/2022/2798217)
Supplement: Supplementary Materials — Table 1: references of Huaier's targets. Table 2: targets of Huaier, GQZ-HJ, HQH, and HSPN. [file 2798217.f1.docx]

Table 1. References of Huaier’s targets

| No | references | targets |
| --- | --- | --- |
| 1 | Kong X, Ding X, Yang Q. Identification of multi-target effects of Huaier aqueous extract via microarray profiling in triple-negative breast cancer cells. Int J Oncol. 2015 May;46(5):2047-56. doi: 10.3892/ijo.2015.2932. Epub 2015 Mar 17. PMID: 25826742. | IL24 |
| 2 | Zou YF, Rong YM, Chen ZX, Shen ZH, Chen X, Tan YX, Weng JR, Huang XM, Lin XT. Effects of Huaier Extract on Ameliorating Colitis-Associated Colorectal Tumorigenesis in Mice. Onco Targets Ther. 2020 Aug 26;13:8691-8704. doi: 10.2147/OTT.S253598. PMID: 32904640; PMCID: PMC7457401. | IL6, TNF |
| 3 | Zhou L, Pan LC, Zheng YG, Zhang XX, Liu ZJ, Meng X, Shi HD, Du GS, He Q. Reduction of FoxP3+ Tregs by an immunosuppressive protocol of rapamycin plus Thymalfasin and Huaier extract predicts positive survival benefits in a rat model of hepatocellular carcinoma. Ann Transl Med. 2020 Apr;8(7):472. doi: 10.21037/atm.2020.03.129. PMID: 32395516; PMCID: PMC7210174. | TGFB1 |
| 4 | Zhong LH, Zhu LY, Zhao YY, Wang W, Lu BL, Wang Y, Cheng Y, Ma YJ. Apoptosis of hepatocarcinoma cells Hepg2 induced by Huaier extract through regulation of HBx and CEACAM1 gene expression. J Biol Regul Homeost Agents. 2018 Nov-Dec;32(6):1389-1398. PMID: 30574743. | CEACAM1, HBx |
| 5 | Yang L, Song Z, Wang X, Yang W, Wang M, Liu H. Huaier extract enhances the treatment efficacy of paclitaxel in breast cancer cells via the NF-κB/IκBα pathway. Oncol Rep. 2017 Dec;38(6):3455-3464. doi: 10.3892/or.2017.6024. Epub 2017 Oct 12. PMID: 29039556. | RELA |
| 6 | Yang A, Fan H, Zhao Y, Chen X, Zhu Z, Zha X, Zhao Y, Chai X, Li J, Tu P, Hu Z. An immune-stimulating proteoglycan from the medicinal mushroom Huaier up-regulates NF-κB and MAPK signaling via Toll-like receptor 4. J Biol Chem. 2019 Feb 22;294(8):2628-2641. doi: 10.1074/jbc.RA118.005477. Epub 2019 Jan 2. PMID: 30602571; PMCID: PMC6393594. | TLR4, PTGS2 |
| 7 | Xu Z, Zheng G, Wang Y, Zhang C, Yu J, Teng F, Lv H, Cheng X. Aqueous Huaier Extract Suppresses Gastric Cancer Metastasis and Epithelial to Mesenchymal Transition by Targeting Twist. J Cancer. 2017 Oct 19;8(18):3876-3886. doi: 10.7150/jca.20380. PMID: 29151976; PMCID: PMC5688942. | TWIST1 |
| 8 | Xie J, Zhuan B, Wang H, Wang Y, Wang X, Yuan Q, Yang Z. Huaier extract suppresses non-small cell lung cancer progression through activating NLRP3-dependent pyroptosis. Anat Rec (Hoboken). 2021 Feb;304(2):291-301. doi: 10.1002/ar.24307. Epub 2019 Nov 22. Retraction in: Anat Rec (Hoboken). 2021 Dec;304(12):2899. PMID: 31692261. | IL18 |
| 9 | Xie HX, Xu ZY, Tang JN, DU YA, Huang L, Yu PF, Cheng XD. Effect of Huaier on the proliferation and apoptosis of human gastric cancer cells through modulation of the PI3K/AKT signaling pathway. Exp Ther Med. 2015 Sep;10(3):1212-1218. doi: 10.3892/etm.2015.2600. Epub 2015 Jun 29. PMID: 26622467; PMCID: PMC4533155. | AKT1, BAD, CASP9, CCNB1, PTEN |
| 10 | Wu T, Chen W, Liu S, Lu H, Wang H, Kong D, Huang X, Kong Q, Ning Y, Lu Z. Huaier suppresses proliferation and induces apoptosis in human pulmonary cancer cells via upregulation of miR-26b-5p. FEBS Lett. 2014 Jun 5;588(12):2107-14. doi: 10.1016/j.febslet.2014.04.044. Epub 2014 May 8. PMID: 24815696. | EZH2 |
| 11 | Wang Y, Lv H, Xu Z, Sun J, Ni Y, Chen Z, Cheng X. Huaier n-butanol extract suppresses proliferation and metastasis of gastric cancer via c-Myc-Bmi1 axis. Sci Rep. 2019 Jan 24;9(1):447. doi: 10.1038/s41598-018-36940-w. PMID: 30679589; PMCID: PMC6346047. | Myc, BMI1 |
| 12 | Wang X, Zhang N, Huo Q, Sun M, Lv S, Yang Q. Huaier aqueous extract suppresses human breast cancer cell proliferation through inhibition of estrogen receptor 伪 signaling. Int J Oncol. 2013 Jul;43(1):321-8. doi: 10.3892/ijo.2013.1947. Epub 2013 May 20. PMID: 23686317. | ESR1 |
| 13 | Wang W, Wang X, Li C, Chen T, Zhang N, Liang Y, Li Y, Zhang H, Liu Y, Song X, Zhao W, Chen B, Wang L, Yang Q. Huaier Suppresses Breast Cancer Progression via linc00339/miR-4656/CSNK2B Signaling Pathway. Front Oncol. 2019 Nov 8;9:1195. doi: 10.3389/fonc.2019.01195. PMID: 31781497; PMCID: PMC6857111. | HDAC3, CSNK2B, CCND3, SRM, UBE2D4, QARS1 |
| 14 | Wang L, Yu Z, Wei C, Zhang L, Song H, Chen B, Yang Q. Huaier aqueous extract protects against dextran sulfate sodium-induced experimental colitis in mice by inhibiting NLRP3 inflammasome activation. Oncotarget. 2017 May 16;8(20):32937-32945. doi: 10.18632/oncotarget.16513. PMID: 28380426; PMCID: PMC5464840. | IL1B, CDKN2A, TP53 |
| 15 | Sun Y, Sun T, Wang F, Zhang J, Li C, Chen X, Li Q, Sun S. A polysaccharide from the fungi of Huaier exhibits anti-tumor potential and immunomodulatory effects. Carbohydr Polym. 2013 Jan 30;92(1):577-82. doi: 10.1016/j.carbpol.2012.09.006. Epub 2012 Sep 13. PMID: 23218338. | NOS2 |
| 16 | Shan L, Li Y, Jiang H, Tao Y, Qian Z, Li L, Cai F, Ma L, Yu Y. Huaier Restrains Proliferative and Migratory Potential of Hepatocellular Carcinoma Cells Partially Through Decreased Yes-Associated Protein 1. J Cancer. 2017 Nov 6;8(19):4087-4097. doi: 10.7150/jca.21018. PMID: 29187885; PMCID: PMC5706012. | YAP1 |
| 17 | Qu P, Zhou F, Tan LF, Wang ZJ, Wang ML, Jin RM, Han J. [Effect of Huaier Aqueous Extract Combined with Routine Chemo-therapeutic Drugs on Human Acute Lymphoblastic Leukemia Cells Nalm-6 and Sup-B15]. Zhongguo Shi Yan Xue Ye Xue Za Zhi. 2020 Oct;28(5):1451-1458. Chinese. doi: 10.19746/j.cnki.issn.1009-2137.2020.05.004. PMID: 33067936. | CASP3 |
| 18 | Pan J, Yang C, Jiang Z, Huang J. *Trametes robiniophila* Murr: a traditional Chinese medicine with potent anti-tumor effects. Cancer Manag Res. 2019 Feb 14;11:1541-1549. doi: 10.2147/CMAR.S193174. PMID: 30863164; PMCID: PMC6389013. | TCP11L2, MAPK, IFNGR1 |
| 19 | Niu Y, Shan L, Gao H, Zhang C, Qian Z, Wang Z, Xu X, Zhang X, Wang J, Ma L, Chen L, Yu Y. Huaier Suppresses the Hepatocellular Carcinoma Cell Cycle by Regulating Minichromosome Maintenance Proteins. Onco Targets Ther. 2020 Nov 20;13:12015-12025. doi: 10.2147/OTT.S279723. PMID: 33244243; PMCID: PMC7685376. | CDK9, MCM2, MCM3, MCM4, MCM5, MCM6, MCM7, CCNE1 |
| 20 | Liu Z, Liu C, Yan K, Liu J, Fang Z, Fan Y. Huaier Extract Inhibits Prostate Cancer Growth *via*Targeting AR/AR-V7 Pathway. Front Oncol. 2021 Feb 23;11:615568. doi: 10.3389/fonc.2021.615568. PMID: 33708629; PMCID: PMC7940541. | AR |
| 21 | Liu X, Liu L, Chen K, Sun L, Li W, Zhang S. Huaier shows anti-cancer activities by inhibition of cell growth, migration and energy metabolism in lung cancer through PI3K/AKT/HIF-1伪 pathway. J Cell Mol Med. 2021 Feb;25(4):2228-2237. doi: 10.1111/jcmm.16215. Epub 2020 Dec 30. PMID: 33377619; PMCID: PMC7882940. | GLUT1, HIF1A |
| 22 | Li Y, Qi W, Song X, Lv S, Zhang H, Yang Q. Huaier extract suppresses breast cancer via regulating tumor-associated macrophages. Sci Rep. 2016 Feb 1;6:20049. doi: 10.1038/srep20049. PMID: 26831282; PMCID: PMC4735520. | MMP2, MMP9, VEGF |
| 23 | Li C, Wu X, Zhang H, Yang G, Hao M, Sheng S, Sun Y, Long J, Hu C, Sun X, Li L, Zheng J. A Huaier polysaccharide inhibits hepatocellular carcinoma growth and metastasis. Tumour Biol. 2015 Mar;36(3):1739-45. doi: 10.1007/s13277-014-2775-2. Epub 2014 Nov 6. PMID: 25374064. | IL10 |
| 24 | Kong X, Ding X, Yang Q. Identification of multi-target effects of Huaier aqueous extract via microarray profiling in triple-negative breast cancer cells. Int J Oncol. 2015 May;46(5):2047-56. doi: 10.3892/ijo.2015.2932. Epub 2015 Mar 17. PMID: 25826742. | F3, CLEC2B, LIPH, ID1, MMP3,MPP4, MAPK1 |
| 25 | Kaleem M, Perwaiz M, Nur SM, Abdulrahman AO, Ahmad W, Al-Abbasi FA, Kumar V, Kamal MA, Anwar F. Epigenetics of Triple-Negative Breast Cancer via Natural Compounds. Curr Med Chem. 2022 Mar 4;29(8):1436-1458. doi: 10.2174/0929867328666210707165530. PMID: 34238140. | IMP3, KLK12, NEK2, GD3S |
| 26 | Hu Z, Yang A, Fan H, Wang Y, Zhao Y, Zha X, Zhang H, Tu P. Huaier aqueous extract sensitizes cells to rapamycin and cisplatin through activating mTOR signaling. J Ethnopharmacol. 2016 Jun 20;186:143-150. doi: 10.1016/j.jep.2016.03.069. Epub 2016 Apr 1. PMID: 27045863. | MTOR |
| 27 | Fu Z, Ma K, Dong B, Zhao C, Che C, Dong C, Zhang R, Wang H, Wang X, Liang R. The synergistic antitumor effect of Huaier combined with 5-Florouracil in human cholangiocarcinoma cells. BMC Complement Altern Med. 2019 Aug 7;19(1):203. doi: 10.1186/s12906-019-2614-5. PMID: 31391034; PMCID: PMC6686517. | CCND1 |
| 28 | Chen Y, Wu H, Wang X, Wang C, Gan L, Zhu J, Tong J, Li Z. Huaier Granule extract inhibit the proliferation and metastasis of lung cancer cells through down-regulation of MTDH, JAK2/STAT3 and MAPK signaling pathways. Biomed Pharmacother. 2018 May;101:311-321. doi: 10.1016/j.biopha.2018.02.028. Epub 2018 Mar 22. PMID: 29499405. | JAK2, MTDH, STAT3, DARC, CXCL1, CXCL8, CCL2, IL8 |
| 29 | Wang Wei.[Construction of Molecular Regulation Network of Huaier in Breast cancer and Functional Analysis of Linc00339].SHANDONG UNIVERSITY.2020.DOI:10.27272/d.cnki.gshdu.2020.000226. | GRPEL1 |
| 30 | Liu Zhengfang.[Genomic hallmarks of prostate cancer and the mechanism of huaier inhibiting prostate cancer progression].SHANDONG UNIVERSITY.2021.DOI:10.27272/d.cnki.gshdu.2021.000220. | CDH1 |

Table 2. Targets of Huaier, GQZ-HJ, HQH and HSPN

| No | Huaier | GQZ-HJ | HQH | HSPN |
| --- | --- | --- | --- | --- |
| 1 | IL24 | ADRB2 | IL24 | MPO |
| 2 | IMP3 | AK1C3 | IMP3 | C2 |
| 3 | DARC | ADORA1 | DARC | VCAM1 |
| 4 | F3 | ADORA2B | F3 | IFNG |
| 5 | CLEC2B | ADA | CLEC2B | ICAM1 |
| 6 | TLR4 | ABCB1 | TLR4 | ACE |
| 7 | CDK9 | ABL1 | CDK9 | C3 |
| 8 | JAK2 | AKR1A1 | JAK2 | TNFSF4 |
| 9 | KLK12 | AKR1B1 | KLK12 | CD79A |
| 10 | HDAC3 | ADRA2B | HDAC3 | SELP |
| 11 | LIPH | AKR1B10 | LIPH | IL4 |
| 12 | CDKN2A | ACHE | CDKN2A | ALB |
| 13 | CXCL1 | ADH1C | CXCL1 | PRTN3 |
| 14 | GLUT1 | ADORA3 | GLUT1 | CFH |
| 15 | AR | ABCC1 | AR | C4A |
| 16 | CSNK2B | ADA17 | CSNK2B | VWF |
| 17 | CEACAM1 | ACP1 | CEACAM1 | MEFV |
| 18 | ID1 | ADRA2C | ID1 | F2 |
| 19 | CCND3 | ABCG2 | CCND3 | CRP |
| 20 | GRPEL1 | ADRA1D | GRPEL1 | C4B |
| 21 | HBx | ADRA2A | HBx | THBD |
| 22 | EZH2 | ADORA2A | EZH2 | ICOSLG |
| 23 | MAPK | AKR1C1 | MAPK | CFB |
| 24 | CXCL8 | ADK | CXCL8 | PTPN22 |
| 25 | MCM2 | AKR1C2 | MCM2 | C1GALT1 |
| 26 | MCM3 | AKR1C3 | MCM3 | RNASE3 |
| 27 | MCM4 | AKR1C4 | MCM4 | CFHR2 |
| 28 | MCM5 | ALDH2 | MCM5 | IL17A |
| 29 | MCM6 | ALDR | MCM6 | SELE |
| 30 | MCM7 | ALK | MCM7 | LTC4S |
| 31 | MMP2 | ALOX12 | MMP2 | VEGFA |
| 32 | MMP3 | ALOX15 | MMP3 | C1GALT1C1 |
| 33 | MMP9 | ALOX5 | MMP9 | FCAR |
| 34 | MPP4 | ALOX5AP | MPP4 | AGT |
| 35 | MTDH | AMPM2 | MTDH | IL1RN |
| 36 | NEK2 | AMY1A | NEK2 | MEST |
| 37 | SRM | AMY2A | SRM | SERPINA3 |
| 38 | STAT3 | ANDR | STAT3 | MIR98 |
| 39 | TCP11L2 | ANXA5 | TCP11L2 | CD40LG |
| 40 | TGFB1 | APP | TGFB1 | AGTR1 |
| 41 | UBE2D4 | ATIC | UBE2D4 | HMGB1 |
| 42 | VEGF | ATP12A | VEGF | TLR2 |
| 43 | YAP1 | AURKA | YAP1 | HAVCR1 |
| 44 | CCNE1 | AURKB | CCNE1 | SERPINE1 |
| 45 | GD3S | AVPR1A | GD3S | CCL5 |
| 46 | Myc | AVPR2 | Myc | CXCR3 |
| 47 | IL1B | AXL | IL1B | HLA-A |
| 48 | RELA | BACE1 | RELA | IL5 |
| 49 | AKT1 | BCHE | AKT1 | IL1R1 |
| 50 | BAD | BCL2L1 | BAD | PAX2 |
| 51 | BMI1 | BIRC7 | BMI1 | SERPINA1 |
| 52 | CASP3 | BMP2 | CASP3 | HMOX1 |
| 53 | CASP9 | BRAF | CASP9 | MIR21 |
| 54 | CCL2 | BTK | CCL2 | IL1A |
| 55 | PTGS2 | CA1 | PTGS2 | HLA-B |
| 56 | CCNB1 | CA12 | CCNB1 | MBL2 |
| 57 | CCND1 | CA13 | CCND1 | TNFRSF4 |
| 58 | CDH1 | CA14 | CDH1 | MTHFR |
| 59 | ESR1 | CA2 | EEF2K | SCGB1A1 |
| 60 | MAPK1 | CA3 | MAPK1 | CXCL16 |
| 61 | HIF1A | CA4 | HIF1A | IL17F |
| 62 | IFNGR1 | CA5A | IFNGR1 | EDN1 |
| 63 | IL10 | CA5B | IL10 | IGHE |
| 64 | IL18 | CA6 | IL18 | IL1RAPL2 |
| 65 | IL6 | CA7 | IL6 | CCL11 |
| 66 | TP53 | CA9 | TP53 | CCL17 |
| 67 | IL8 | CACNA2D1 | IL8 | MASP1 |
| 68 | NOS2 | CACNA2D2 | NOS2 | C5AR1 |
| 69 | MTOR | CAH2 | MTOR | CX3CL1 |
| 70 | PNMT | CALCRL | PNMT | SOD1 |
| 71 | QARS1 | CALM | QARS1 | IGF1 |
| 72 | TNF | CAMK2B | TNF | ILK |
| 73 | TWIST1 | CASP7 | TWIST1 | SELL |
| 74 |  | CASR | ABCB1 | CYP21A2 |
| 75 |  | CATK | ABCC1 | HADHA |
| 76 |  | CBR1 | ABCG2 | ACADVL |
| 77 |  | CCNA2 | ABL1 | PRSS1 |
| 78 |  | CCNB3 | ACHE | MIR152 |
| 79 |  | CCR1 | ACP1 | C1D |
| 80 |  | CDC25A | ADA | IGHA1 |
| 81 |  | CDC25B | ADA17 | NOS3 |
| 82 |  | CDC7 | ADH1C | HSP90B2P |
| 83 |  | CDK1 | ADK | IGAN1 |
| 84 |  | CDK2 | ADORA1 | CD40 |
| 85 |  | CDK5R1 | ADORA2A | NPHS2 |
| 86 |  | CDK6 | ADORA2B | TFRC |
| 87 |  | CDK8 | ADORA3 | ESR1 |
| 88 |  | CES1 | ADRA1D | NR3C1 |
| 89 |  | CES2 | ADRA2A | MIR29B1 |
| 90 |  | CFAD | ADRA2B | MIR29B2 |
| 91 |  | CFD | ADRA2C | MIF |
| 92 |  | CFTR | ADRB2 | TINAGL1 |
| 93 |  | CHEK1 | AK1C3 | FAN1 |
| 94 |  | CHIA | AKR1A1 | F5 |
| 95 |  | CHIT1 | AKR1B1 | PROC |
| 96 |  | CHK1 | AKR1B10 | FCGR2C |
| 97 |  | CHRM1 | AKR1C1 | GLB1 |
| 98 |  | CHRM2 | AKR1C2 | INF2 |
| 99 |  | CHRM3 | AKR1C3 | MUC20 |
| 100 |  | CHRM4 | AKR1C4 | CD109 |
| 101 |  | CHRM5 | ALDH2 | MRPL3 |
| 102 |  | CHRNA3 | ALDR | IFIH1 |
| 103 |  | CHRNA7 | ALK | TINAG |
| 104 |  | CHRNB3 | ALOX12 | TREX1 |
| 105 |  | CLK1 | ALOX15 | LRBA |
| 106 |  | CNR1 | ALOX5 | SLC2A9 |
| 107 |  | CNR2 | ALOX5AP | TNFRSF13B |
| 108 |  | CP2C8 | AMPM2 | TFR2 |
| 109 |  | CP2C9 | AMY1A | ADAMTS13 |
| 110 |  | CRABP2 | AMY2A | SERPINB7 |
| 111 |  | CRHR1 | ANDR | FOXC2 |
| 112 |  | CSNK2A1 | ANXA5 | CD151 |
| 113 |  | CTNA1 | APP | SIPA1 |
| 114 |  | CTRC | ATIC | PHYH |
| 115 |  | CXCR1 | ATP12A | CCR5 |
| 116 |  | CYP17A1 | AURKA | FKBP4 |
| 117 |  | CYP19A1 | AURKB | BAK1 |
| 118 |  | CYP1B1 | AVPR1A | GAS6 |
| 119 |  | CYP24A1 | AVPR2 | LRP2 |
| 120 |  | CYP2C19 | AXL | BAX |
| 121 |  | CYP2C9 | BACE1 | MTTF |
| 122 |  | CYP3A4 | BCHE | MTATP8 |
| 123 |  | CYP51A1 | BCL2L1 | COL4A5 |
| 124 |  | DAPK1 | BIRC7 | WAS |
| 125 |  | DGAT1 | BMP2 | INVS |
| 126 |  | DHCR7 | BRAF | C6 |
| 127 |  | DHI1 | BTK | MAF |
| 128 |  | DNM1 | CA1 | PROS1 |
| 129 |  | DNMT1 | CA12 | TTR |
| 130 |  | DPEP1 | CA13 | ITGB3 |
| 131 |  | DPP4 | CA14 | ACP5 |
| 132 |  | DPP8 | CA2 | RET |
| 133 |  | DPP9 | CA3 | ETS1 |
| 134 |  | DRD2 | CA4 | MYH9 |
| 135 |  | DRD3 | CA5A | PTPRC |
| 136 |  | DRD4 | CA5B | FCGR2A |
| 137 |  | DYRK1B | CA6 | CFD |
| 138 |  | EDNRA | CA7 | C5 |
| 139 |  | EGFR | CA9 | COL4A4 |
| 140 |  | ELNE | CACNA2D1 | COL4A3 |
| 141 |  | EPAS1 | CACNA2D2 | CD80 |
| 142 |  | EPCR | CAH2 | APOA1 |
| 143 |  | EPHA2 | CALCRL | CD72 |
| 144 |  | EPHB4 | CALM | LRPAP1 |
| 145 |  | EPHX1 | CAMK2B | TLR4 |
| 146 |  | EPHX2 | CASP7 | CXCL8 |
| 147 |  | ERBB4 | CASR | MMP9 |
| 148 |  | ERG7 | CATK | STAT3 |
| 149 |  | ESR1 | CBR1 | TGFB1 |
| 150 |  | ESR2 | CCNA2 | IL1B |
| 151 |  | ESRRA | CCNB3 | RELA |
| 152 |  | ESRRB | CCR1 | AKT1 |
| 153 |  | F10 | CDC25A | CCL2 |
| 154 |  | F2 | CDC25B | IL10 |
| 155 |  | F2R | CDC7 | IL18 |
| 156 |  | FA11 | CDK1 | IL6 |
| 157 |  | FA7 | CDK2 | TNF |
| 158 |  | FAAH | CDK5R1 |  |
| 159 |  | FABP1 | CDK6 |  |
| 160 |  | FABP3 | CDK8 |  |
| 161 |  | FABP4 | CES1 |  |
| 162 |  | FABP5 | CES2 |  |
| 163 |  | FABP6 | CFAD |  |
| 164 |  | FABP7 | CFD |  |
| 165 |  | FABPH | CFTR |  |
| 166 |  | FASN | CHEK1 |  |
| 167 |  | FDFT1 | CHIA |  |
| 168 |  | FECH | CHIT1 |  |
| 169 |  | FFAR1 | CHK1 |  |
| 170 |  | FGFR1 | CHRM1 |  |
| 171 |  | FKB1A | CHRM2 |  |
| 172 |  | FKB1B | CHRM3 |  |
| 173 |  | FLT3 | CHRM4 |  |
| 174 |  | FNTA | CHRM5 |  |
| 175 |  | FNTA FNTB | CHRNA3 |  |
| 176 |  | FOLH1 | CHRNA7 |  |
| 177 |  | FUCA1 | CHRNB3 |  |
| 178 |  | FUT4 | CLK1 |  |
| 179 |  | FUT7 | CNR1 |  |
| 180 |  | G6PD | CNR2 |  |
| 181 |  | GAA | CP2C8 |  |
| 182 |  | GLO1 | CP2C9 |  |
| 183 |  | GLRA1 | CRABP2 |  |
| 184 |  | GM2A | CRHR1 |  |
| 185 |  | GNPDA1 | CSNK2A1 |  |
| 186 |  | GPR35 | CTNA1 |  |
| 187 |  | GRIA2 | CTRC |  |
| 188 |  | GRIN2A | CXCR1 |  |
| 189 |  | GRK6 | CYP17A1 |  |
| 190 |  | GRM2 | CYP19A1 |  |
| 191 |  | GRM5 | CYP1B1 |  |
| 192 |  | GSK3A | CYP24A1 |  |
| 193 |  | GSK3B | CYP2C19 |  |
| 194 |  | GSTA1 | CYP2C9 |  |
| 195 |  | GSTP1 | CYP3A4 |  |
| 196 |  | GUSB | CYP51A1 |  |
| 197 |  | HCRTR1 | DAPK1 |  |
| 198 |  | HCRTR2 | DGAT1 |  |
| 199 |  | HDAC8 | DHCR7 |  |
| 200 |  | HMGCR | DHI1 |  |
| 201 |  | HNF4G | DNM1 |  |
| 202 |  | HNMT | DNMT1 |  |
| 203 |  | HPGDS | DPEP1 |  |
| 204 |  | HPN | DPP4 |  |
| 205 |  | HRAS | DPP8 |  |
| 206 |  | HRH1 | DPP9 |  |
| 207 |  | HRH3 | DRD2 |  |
| 208 |  | HRH4 | DRD3 |  |
| 209 |  | HS90A | DRD4 |  |
| 210 |  | HSD11B1 | DYRK1B |  |
| 211 |  | HSD11B2 | EDNRA |  |
| 212 |  | HSD17B1 | EGFR |  |
| 213 |  | HSD17B14 | ELNE |  |
| 214 |  | HSD17B2 | EPAS1 |  |
| 215 |  | HSP90AB1 | EPCR |  |
| 216 |  | HTR1A | EPHA2 |  |
| 217 |  | HTR1D | EPHB4 |  |
| 218 |  | HTR1F | EPHX1 |  |
| 219 |  | HTR2A | EPHX2 |  |
| 220 |  | HTR2B | ERBB4 |  |
| 221 |  | HTR2C | ERG7 |  |
| 222 |  | HTR6 | ESR1 |  |
| 223 |  | HXK4 | ESR2 |  |
| 224 |  | IDH1 | ESRRA |  |
| 225 |  | IGF1 | ESRRB |  |
| 226 |  | IGF1R | F10 |  |
| 227 |  | IGFBP3 | F2 |  |
| 228 |  | IKBKB | F2R |  |
| 229 |  | IL2 | FA11 |  |
| 230 |  | IL6ST | FA7 |  |
| 231 |  | ILK | FAAH |  |
| 232 |  | INCENP | FABP1 |  |
| 233 |  | INSR | FABP3 |  |
| 234 |  | ITK | FABP4 |  |
| 235 |  | ITK1 | FABP5 |  |
| 236 |  | JAK3 | FABP6 |  |
| 237 |  | KCNA3 | FABP7 |  |
| 238 |  | KCNH2 | FABPH |  |
| 239 |  | KDM4E | FASN |  |
| 240 |  | KDR | FDFT1 |  |
| 241 |  | KIT | FECH |  |
| 242 |  | KLK1 | FFAR1 |  |
| 243 |  | KLK2 | FGFR1 |  |
| 244 |  | KPCT | FKB1A |  |
| 245 |  | LCK | FKB1B |  |
| 246 |  | LGALS3 | FLT3 |  |
| 247 |  | LGALS4 | FNTA |  |
| 248 |  | LGALS8 | FNTA FNTB | |
| 249 |  | LGALS9 | FOLH1 |  |
| 250 |  | LSS | FUCA1 |  |
| 251 |  | MAG | FUT4 |  |
| 252 |  | MAOA | FUT7 |  |
| 253 |  | MAOB | G6PD |  |
| 254 |  | MAP3K9 | GAA |  |
| 255 |  | MAPK10 | GLO1 |  |
| 256 |  | MAPK14 | GLRA1 |  |
| 257 |  | MAPK3 | GM2A |  |
| 258 |  | MAPK8 | GNPDA1 |  |
| 259 |  | MAPK9 | GPR35 |  |
| 260 |  | MAPKAPK2 | GRIA2 |  |
| 261 |  | MAPT | GRIN2A |  |
| 262 |  | MCL1 | GRK6 |  |
| 263 |  | MCR | GRM2 |  |
| 264 |  | MDM2 | GRM5 |  |
| 265 |  | MDM4 | GSK3A |  |
| 266 |  | MERTK | GSK3B |  |
| 267 |  | MET | GSTA1 |  |
| 268 |  | METAP1 | GSTP1 |  |
| 269 |  | METAP2 | GUSB |  |
| 270 |  | MGAM | HCRTR1 |  |
| 271 |  | MGLL | HCRTR2 |  |
| 272 |  | MIF | HDAC8 |  |
| 273 |  | MK08 | HMGCR |  |
| 274 |  | MK10 | HNF4G |  |
| 275 |  | MMP1 | HNMT |  |
| 276 |  | MMP12 | HPGDS |  |
| 277 |  | MMP13 | HPN |  |
| 278 |  | MMP14 | HRAS |  |
| 279 |  | MMP7 | HRH1 |  |
| 280 |  | MMP8 | HRH3 |  |
| 281 |  | MP2K1 | HRH4 |  |
| 282 |  | MPO | HS90A |  |
| 283 |  | MTAP | HSD11B1 |  |
| 284 |  | MTNR1A | HSD11B2 |  |
| 285 |  | MTNR1B | HSD17B1 |  |
| 286 |  | MYLK | HSD17B14 |  |
| 287 |  | NEK6 | HSD17B2 |  |
| 288 |  | NGAL | HSP90AB1 |  |
| 289 |  | NOX4 | HTR1A |  |
| 290 |  | NPC1L1 | HTR1D |  |
| 291 |  | NPY1R | HTR1F |  |
| 292 |  | NPY5R | HTR2A |  |
| 293 |  | NQO1 | HTR2B |  |
| 294 |  | NR1H2 | HTR2C |  |
| 295 |  | NR1H3 | HTR6 |  |
| 296 |  | NR1H4 | HXK4 |  |
| 297 |  | NR1I2 | IDH1 |  |
| 298 |  | NR1I3 | IGF1 |  |
| 299 |  | NR3C1 | IGF1R |  |
| 300 |  | NR3C2 | IGFBP3 |  |
| 301 |  | NTRK1 | IKBKB |  |
| 302 |  | NTRK2 | IL2 |  |
| 303 |  | NUAK1 | IL6ST |  |
| 304 |  | OGA | ILK |  |
| 305 |  | OPRD1 | INCENP |  |
| 306 |  | OPRK1 | INSR |  |
| 307 |  | OPRL1 | ITK |  |
| 308 |  | OPRM1 | ITK1 |  |
| 309 |  | OXTR | JAK3 |  |
| 310 |  | P04745 | KCNA3 |  |
| 311 |  | P2RX3 | KCNH2 |  |
| 312 |  | PABPC1 | KDM4E |  |
| 313 |  | PARP1 | KDR |  |
| 314 |  | PCSK7 | KIT |  |
| 315 |  | PCTP | KLK1 |  |
| 316 |  | PDE10A | KLK2 |  |
| 317 |  | PDE1A | KPCT |  |
| 318 |  | PDE1C | LCK |  |
| 319 |  | PDE2A | LGALS3 |  |
| 320 |  | PDE4B | LGALS4 |  |
| 321 |  | PDE4D | LGALS8 |  |
| 322 |  | PDE5A | LGALS9 |  |
| 323 |  | PDE9A | LSS |  |
| 324 |  | PDGFRB | MAG |  |
| 325 |  | PDK2 | MAOA |  |
| 326 |  | PFKFB3 | MAOB |  |
| 327 |  | PGD | MAP3K9 |  |
| 328 |  | PGGT1B | MAPK10 |  |
| 329 |  | PGR | MAPK14 |  |
| 330 |  | PIK3CA | MAPK3 |  |
| 331 |  | PIK3CB | MAPK8 |  |
| 332 |  | PIK3CD | MAPK9 |  |
| 333 |  | PIK3CG | MAPKAPK2 | |
| 334 |  | PIK3R1 | MAPT |  |
| 335 |  | PIM1 | MCL1 |  |
| 336 |  | PK3CG | MCR |  |
| 337 |  | PKN1 | MDM2 |  |
| 338 |  | PLA2G10 | MDM4 |  |
| 339 |  | PLA2G1B | MERTK |  |
| 340 |  | PLA2G2A | MET |  |
| 341 |  | PLA2G5 | METAP1 |  |
| 342 |  | PLAT | METAP2 |  |
| 343 |  | PLAU | MGAM |  |
| 344 |  | PLD1 | MGLL |  |
| 345 |  | PLD2 | MIF |  |
| 346 |  | PLGF | MK08 |  |
| 347 |  | PLK1 | MK10 |  |
| 348 |  | POLA1 | MMP1 |  |
| 349 |  | POLB | MMP12 |  |
| 350 |  | PON1 | MMP13 |  |
| 351 |  | PORCN | MMP14 |  |
| 352 |  | PPARA | MMP7 |  |
| 353 |  | PPARD | MMP8 |  |
| 354 |  | PPARG | MP2K1 |  |
| 355 |  | PPP1CC | MPO |  |
| 356 |  | PPP5C | MTAP |  |
| 357 |  | PREP | MTNR1A |  |
| 358 |  | PRGR | MTNR1B |  |
| 359 |  | PRKACA | MYLK |  |
| 360 |  | PRKCA | NEK6 |  |
| 361 |  | PRKCB | NGAL |  |
| 362 |  | PRKCD | NOX4 |  |
| 363 |  | PRKCE | NPC1L1 |  |
| 364 |  | PRKCG | NPY1R |  |
| 365 |  | PRKCH | NPY5R |  |
| 366 |  | PRKCQ | NQO1 |  |
| 367 |  | PRMT1 | NR1H2 |  |
| 368 |  | PRMT6 | NR1H3 |  |
| 369 |  | PRMT8 | NR1H4 |  |
| 370 |  | PRSS1 | NR1I2 |  |
| 371 |  | PSAP | NR1I3 |  |
| 372 |  | PSEN2 | NR3C1 |  |
| 373 |  | PTGER1 | NR3C2 |  |
| 374 |  | PTGER2 | NTRK1 |  |
| 375 |  | PTGES | NTRK2 |  |
| 376 |  | PTGIR | NUAK1 |  |
| 377 |  | PTGS1 | OGA |  |
| 378 |  | PTK2 | OPRD1 |  |
| 379 |  | PTK2B | OPRK1 |  |
| 380 |  | PTK6 | OPRL1 |  |
| 381 |  | PTN1 | OPRM1 |  |
| 382 |  | PTN11 | OXTR |  |
| 383 |  | PTPN11 | P04745 |  |
| 384 |  | PTPN2 | P2RX3 |  |
| 385 |  | PTPN6 | PABPC1 |  |
| 386 |  | PTPRF | PARP1 |  |
| 387 |  | PTPRS | PCSK7 |  |
| 388 |  | PYGL | PCTP |  |
| 389 |  | PYRD | PDE10A |  |
| 390 |  | RARA | PDE1A |  |
| 391 |  | RARB | PDE1C |  |
| 392 |  | RARG | PDE2A |  |
| 393 |  | RASGRP3 | PDE4B |  |
| 394 |  | REN | PDE4D |  |
| 395 |  | RENI | PDE5A |  |
| 396 |  | RET4 | PDE9A |  |
| 397 |  | ROCK1 | PDGFRB |  |
| 398 |  | ROCK2 | PDK2 |  |
| 399 |  | RORA | PFKFB3 |  |
| 400 |  | RORC | PGD |  |
| 401 |  | RXRA | PGGT1B |  |
| 402 |  | RXRB | PGR |  |
| 403 |  | S10A9 | PIK3CA |  |
| 404 |  | S1PR1 | PIK3CB |  |
| 405 |  | S1PR3 | PIK3CD |  |
| 406 |  | SAE1 | PIK3CG |  |
| 407 |  | SCD | PIK3R1 |  |
| 408 |  | SEC14L2 | PIM1 |  |
| 409 |  | SERPINA6 | PK3CG |  |
| 410 |  | SERPINE1 | PKN1 |  |
| 411 |  | SHBG | PLA2G10 |  |
| 412 |  | SHH | PLA2G1B |  |
| 413 |  | SI | PLA2G2A |  |
| 414 |  | SIGMAR1 | PLA2G5 |  |
| 415 |  | SLC29A1 | PLAT |  |
| 416 |  | SLC5A1 | PLAU |  |
| 417 |  | SLC5A2 | PLD1 |  |
| 418 |  | SLC5A4 | PLD2 |  |
| 419 |  | SLC6A2 | PLGF |  |
| 420 |  | SLC6A3 | PLK1 |  |
| 421 |  | SLC6A4 | POLA1 |  |
| 422 |  | SLC6A9 | POLB |  |
| 423 |  | SMO | PON1 |  |
| 424 |  | SNCA | PORCN |  |
| 425 |  | SOAT1 | PPARA |  |
| 426 |  | SOAT2 | PPARD |  |
| 427 |  | SOD2 | PPARG |  |
| 428 |  | SQLE | PPP1CC |  |
| 429 |  | SRC | PPP5C |  |
| 430 |  | SREBF2 | PREP |  |
| 431 |  | ST3GAL3 | PRGR |  |
| 432 |  | STAT1 | PRKACA |  |
| 433 |  | STS | PRKCA |  |
| 434 |  | SULT2A1 | PRKCB |  |
| 435 |  | SULT2B1 | PRKCD |  |
| 436 |  | SYK | PRKCE |  |
| 437 |  | TACR1 | PRKCG |  |
| 438 |  | TAS2R31 | PRKCH |  |
| 439 |  | TBXAS1 | PRKCQ |  |
| 440 |  | TDP1 | PRMT1 |  |
| 441 |  | TERT | PRMT6 |  |
| 442 |  | TGFB2 | PRMT8 |  |
| 443 |  | THRB | PRSS1 |  |
| 444 |  | TIE2 | PSAP |  |
| 445 |  | TKT | PSEN2 |  |
| 446 |  | TLR9 | PTGER1 |  |
| 447 |  | TNKS | PTGER2 |  |
| 448 |  | TNKS2 | PTGES |  |
| 449 |  | TNNC1 | PTGIR |  |
| 450 |  | TOP1 | PTGS1 |  |
| 451 |  | TOP2A | PTK2 |  |
| 452 |  | TRAPPC3 | PTK2B |  |
| 453 |  | TRPV3 | PTK6 |  |
| 454 |  | TRYB2 | PTN1 |  |
| 455 |  | TTK | PTN11 |  |
| 456 |  | TTPA | PTPN11 |  |
| 457 |  | TTR | PTPN2 |  |
| 458 |  | TYMP | PTPN6 |  |
| 459 |  | TYR | PTPRF |  |
| 460 |  | TYRO3 | PTPRS |  |
| 461 |  | UGT2B7 | PYGL |  |
| 462 |  | VDR | PYRD |  |
| 463 |  | VTDB | RARA |  |
| 464 |  | WEE1 | RARB |  |
| 465 |  | XDH | RARG |  |
| 466 |  | XIAP | RASGRP3 |  |
| 467 |  | ZAP70 | REN |  |
| 468 |  | AKT1 | RENI |  |
| 469 |  | AR | RET4 |  |
| 470 |  | CASP3 | ROCK1 |  |
| 471 |  | CDK9 | ROCK2 |  |
| 472 |  | HIF1A | RORA |  |
| 473 |  | JAK2 | RORC |  |
| 474 |  | MMP2 | RXRA |  |
| 475 |  | MMP3 | RXRB |  |
| 476 |  | MMP9 | S10A9 |  |
| 477 |  | MTOR | S1PR1 |  |
| 478 |  | NEK2 | S1PR3 |  |
| 479 |  | NOS2 | SAE1 |  |
| 480 |  | PNMT | SCD |  |
| 481 |  | PTGS2 | SEC14L2 |  |
| 482 |  | STAT3 | SERPINA6 |  |
| 483 |  |  | SERPINE1 |  |
| 484 |  |  | SHBG |  |
| 485 |  |  | SHH |  |
| 486 |  |  | SI |  |
| 487 |  |  | SIGMAR1 |  |
| 488 |  |  | SLC29A1 |  |
| 489 |  |  | SLC5A1 |  |
| 490 |  |  | SLC5A2 |  |
| 491 |  |  | SLC5A4 |  |
| 492 |  |  | SLC6A2 |  |
| 493 |  |  | SLC6A3 |  |
| 494 |  |  | SLC6A4 |  |
| 495 |  |  | SLC6A9 |  |
| 496 |  |  | SMO |  |
| 497 |  |  | SNCA |  |
| 498 |  |  | SOAT1 |  |
| 499 |  |  | SOAT2 |  |
| 500 |  |  | SOD2 |  |
| 501 |  |  | SQLE |  |
| 502 |  |  | SRC |  |
| 503 |  |  | SREBF2 |  |
| 504 |  |  | ST3GAL3 |  |
| 505 |  |  | STAT1 |  |
| 506 |  |  | STS |  |
| 507 |  |  | SULT2A1 |  |
| 508 |  |  | SULT2B1 |  |
| 509 |  |  | SYK |  |
| 510 |  |  | TACR1 |  |
| 511 |  |  | TAS2R31 |  |
| 512 |  |  | TBXAS1 |  |
| 513 |  |  | TDP1 |  |
| 514 |  |  | TERT |  |
| 515 |  |  | TGFB2 |  |
| 516 |  |  | THRB |  |
| 517 |  |  | TIE2 |  |
| 518 |  |  | TKT |  |
| 519 |  |  | TLR9 |  |
| 520 |  |  | TNKS |  |
| 521 |  |  | TNKS2 |  |
| 522 |  |  | TNNC1 |  |
| 523 |  |  | TOP1 |  |
| 524 |  |  | TOP2A |  |
| 525 |  |  | TRAPPC3 |  |
| 526 |  |  | TRPV3 |  |
| 527 |  |  | TRYB2 |  |
| 528 |  |  | TTK |  |
| 529 |  |  | TTPA |  |
| 530 |  |  | TTR |  |
| 531 |  |  | TYMP |  |
| 532 |  |  | TYR |  |
| 533 |  |  | TYRO3 |  |
| 534 |  |  | UGT2B7 |  |
| 535 |  |  | VDR |  |
| 536 |  |  | VTDB |  |
| 537 |  |  | WEE1 |  |
| 538 |  |  | XDH |  |
| 539 |  |  | XIAP |  |
| 540 |  |  | ZAP70 |  |
